# Transposable elements as a potent source of diverse *cis*-regulatory sequences in mammalian genomes

**DOI:** 10.1098/rstb.2019.0347

**Published:** 2020-02-10

**Authors:** Vasavi Sundaram, Joanna Wysocka

**Affiliations:** 1 European Molecular Biology Laboratory, European Bioinformatics Institute, Wellcome Genome Campus, Hinxton, Cambridge CB10 1SD, UK; 2 Department of Chemical and Systems Biology, Stanford University School of Medicine, Stanford, USA; 3 Department of Developmental Biology, Stanford University School of Medicine, Stanford, USA; 4 Howard Hughes Medical Institute, Stanford University School of Medicine, Stanford, USA

**Keywords:** transcription factor binding, transposons, enhancers, hourglass model and gene regulation

## Abstract

Eukaryotic gene regulation is mediated by *cis*-regulatory elements, which are embedded within the vast non-coding genomic space and recognized by the transcription factors in a sequence- and context-dependent manner. A large proportion of eukaryotic genomes, including at least half of the human genome, are composed of transposable elements (TEs), which in their ancestral form carried their own *cis*-regulatory sequences able to exploit the host *trans* environment to promote TE transcription and facilitate transposition. Although not all present-day TE copies have retained this regulatory function, the preexisting regulatory potential of TEs can provide a rich source of *cis*-regulatory innovation for the host. Here, we review recent evidence documenting diverse contributions of TE sequences to gene regulation by functioning as enhancers, promoters, silencers and boundary elements. We discuss how TE-derived enhancer sequences can rapidly facilitate changes in existing gene regulatory networks and mediate species- and cell-type-specific regulatory innovations, and we postulate a unique contribution of TEs to species-specific gene expression divergence in pluripotency and early embryogenesis. With advances in genome-wide technologies and analyses, systematic investigation of TEs' *cis*-regulatory potential is now possible and our understanding of the biological impact of genomic TEs is increasing.

This article is part of a discussion meeting issue ‘Crossroads between transposons and gene regulation’.

## 1. Introduction

Following Barbara McClintock's foundational maize kernel experiments in which transposable elements (TEs) were first discovered [1], Davidson & Britten [2] postulated a possible role of repetitive sequences and TEs in gene regulation. These prescient early predictions have now found abundant experimental support. While early work documented individual instances of TE co-option for regulatory functions, systematic analysis of TE contribution to gene regulation was for a long time limited by technological challenges. The repetitiveness of TE sequences across a genome made it difficult to precisely identify mapping locations of next-generation sequencing (NGS) data from TEs and precluded systematic perturbative experiments. However, significant advances in computational software and longer sequencing reads now facilitate mapping of individual TEs in the genome. The vast majority of TEs have lost their ability to actively transpose (fewer than 1% of human TEs are capable of transposition, reviewed in [3]), and over the course of evolutionary time have accumulated mutations that increase the uniqueness of each TE and enable higher recovery of mapped NGS reads. Therefore, large-scale epigenomic and transcriptomic studies can now include the previously ignored approximately 50% (and perhaps up to approx. 70% [4]) of the genome derived from TEs, allowing scientists to systematically identify candidate TEs that escape epigenetic silencing and gain regulatory signatures. Additionally, high-throughput genomic technologies (such as massively parallel reporter assays or CRISPR editing/activation/interference) for quantifying regulatory potential of specific sequences, deleting genomic regions or perturbing their function now enable us to determine functional roles of TEs in gene regulation and genome organization. These technological advancements have led to a recent explosion of studies documenting TE contribution to gene regulation via various *cis*-regulatory modes, which we discuss here with a focus on mammalian gene regulation.

## 2. Transcription factor binding sites contributed by transposable elements

Transcription factors (TF) are proteins that regulate gene expression by binding to DNA at specific sequence motifs. Chromatin immunoprecipitation (ChIP) maps of genome-wide TF occupancy demonstrate abundant binding of diverse TFs (including STAT1 [5], TP53 [6], OCT4, NANOG and CTCF [7–9]) within TE sequences. Large-scale genome-wide analyses of 26 TFs by using sequencing (ChIP-seq) in human and mouse cells [10] revealed that up to 40% (minimum: 2%, average: 20%) of TF-binding events are derived from TEs [11]. TE-derived TF-binding sites were widespread—every TF assayed had some fraction of binding events in TEs. Moreover, waves of expansions of various TE families at different evolutionary times appear to coincide with the expansion of target repertoire for specific TFs [12]. A majority of the TEs that were bound by the given TF also contained DNA sequence motifs for the TF, suggesting direct, sequence-dependent TF binding to TEs. Transcription factors with more binding locations in the genome also had higher fractions of binding to TEs [6]. This increase in the number of TF-binding locations mediated by TEs could be associated with an increase in the TF's gene target repertoire and broader impact on gene expression, although the latter notion remains to be functionally assessed [13]. This supports the idea that TEs could contribute to coordinated regulation in the genome by spreading DNA substrate for TF binding across the genome [2].

**Figure 1.**
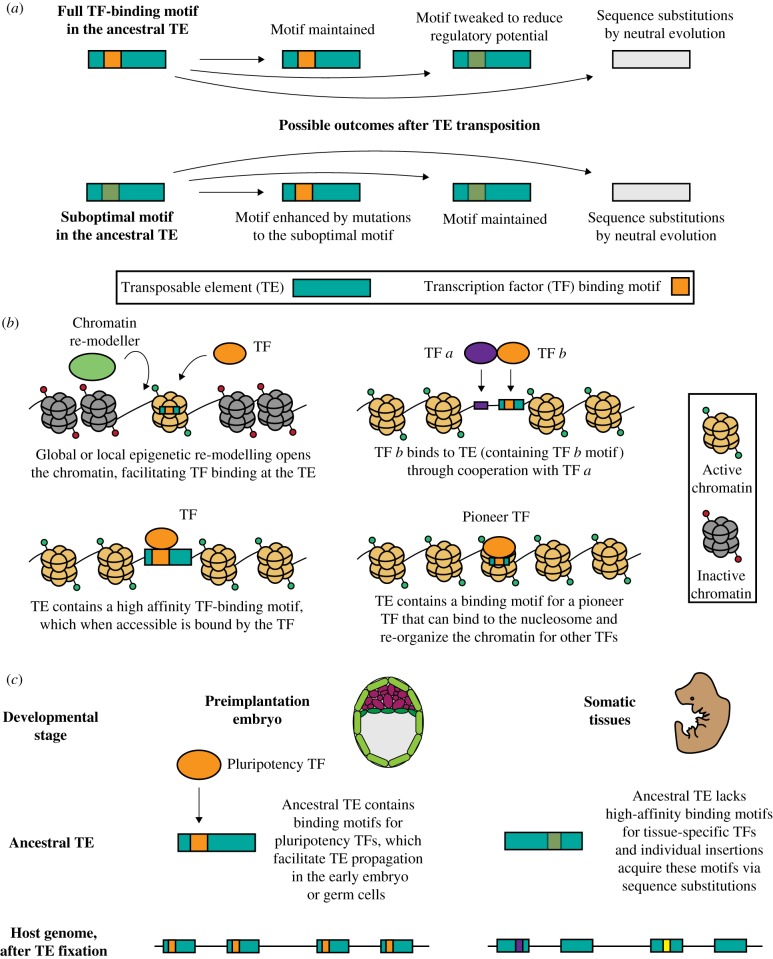
TF binding to TEs. (*a*) There are various possible outcomes from transposition of the ancestral TE (teal rectangles) that leads to variation in TF-binding motifs (orange motifs) observed in present-day TEs. When ancestral TEs contain functional TF-binding motifs (upper panel), they can spread these motifs across the genome, which might be co-opted and maintained, modified or lost by neutral substitution. Alternatively, ancestral TEs might serve as a substrate for the evolution of new or enhanced TF-binding motifs (lower panel). (*b*) TF binding is not only dependent on sequence but also on chromatin context. (Clockwise from top-left) TF binding can occur after chromatin re-modelling, through cooperation with another TF, through the binding of a pioneer TF to the nucleosome or through direct binding to a strong motif. (*c*) Differences in the TFs that bind TEs correspond to developmental stages. Preimplantation embryos express pluripotency TFs that can bind to the ancestral TE and also permit en masse TE entry into the genome. Alternatively, in somatic tissues, TEs might not have somatic TF-binding motifs but could evolve them via neutral substitutions. Ancestral TEs can contain suboptimal TF-binding motifs that become a bona fide binding site for TFs in somatic tissues, through a few nucleotide modifications.

TE-derived TF-binding sites are non-randomly distributed across TE subfamilies. Specific TE subfamilies enrich for the binding of distinct TFs [11]. Based on 26 TFs assayed by ChIP-seq in human and mouse, there are 710 TE subfamilies that enrich for binding of at least one TF. In the genome, TE subfamilies have several hundred (up to a few thousands) of individual insertions, most of which accumulate substitutions through genetic drift (figure 1*a*). It has been widely suggested that TEs form the substrate for TF-binding site evolution and innovation [14]. However, even for TE subfamilies enriched for specific TF motifs, not every genomic copy of a TE can be effectively bound by those TFs for several reasons. First, most TEs are epigenetically silenced and thus can be accessed by TFs only under conditions in which this repression can be overcome by either the high affinity of a TF for the TE-derived binding motif, its ability to function as a pioneer factor that can directly bind nucleosomal DNA, cooperativity with other TFs or global or local epigenetic changes [15–18] (figure 1*b*). Second, a certain fraction of elements within a TE subfamily might contain a given TF-binding motif, while many others might have lost it via genetic drift [6,11] (figure 1*a*). Alternatively, uneven distribution of TF binding within a TE subfamily may arise, because only a few actively transposing TE copies acquired the motif (figure 1*a*), which they then distributed throughout the genome. For example, recent work [19] on the *Mus caroli* genome revealed a unique variant of CTCF-binding sites (motif variation: 18C→T) contributed by the species-specific expansion of rodent B2 SINE (B2_Mm1; Short INterspersed Elements—Class I, non-autonomous TEs). Finally, individual insertions within a TE subfamily may acquire TF-binding activity via mutations that complete an already present suboptimal motif (figure 1*a*). All aforementioned mechanisms contribute to the observed non-uniform binding of TFs within TE subfamilies.

The majority of TE-derived TF-binding events are species-specific and are driven by the expansion of different TE subfamilies at different evolutionary timepoints [6,8,9,11]. Species-specific effects of TEs on gene regulation are demonstrated for various TFs, including pluripotency factors (OCT4, NANOG), CTCF, tumour-suppressor TP53, STAT1 (involved in immune response [20]) and pregnancy-associated factors regulated by cAMP [21]. TEs are widely involved in placental development in mammals including the exaptation of *syncytin* [22–25] and the contribution of placental enhancers [26]. A DNA transposon (Class II TEs), MER20, is associated with approximately 13% of the differentially expressed endometrial genes across mammals and is thought to have re-modelled the placental gene regulatory network by recruiting cAMP signalling to endometrial cells. MER20 contains binding sites for various TFs, which also acquire chromatin signatures associated with functional regulatory elements [21]. In addition, ancient mammalian TEs are enriched for binding sites of TFs associated with hormone responsiveness and involved in specifying the endometrial cellular identity [26]. TE-derived TF-binding sites could also provide robustness to gene regulation programmes by increasing the redundancy in the network for TF binding. Alternatively, recent analyses of cancer samples reveal that TF-binding sites in TEs can also drive tumour formation and prognosis [27]. Taken together, there is broad evidence for TEs being bound by TFs and impacting gene regulation in direct and indirect ways.

## 3. Diversity of *cis*-regulatory modules encoded by transposable elements

*Cis*-regulatory modules represent clusters of TF binding, and many TE sequences are bound by multiple TFs. For example, rodent endogenous retroviruses (ERVs), including a mouse-specific RLTR13D5, contribute species-specific enhancers to placental development in trophoblast stem cells (TSCs) and are bound by critical trophoblast TFs, including Eomes, Cdx2 and Elf5 [28]. Similarly, pluripotency factors co-bind certain mouse-specific LTRs (i.e. long terminal repeats—Class I, autonomous TEs) and are capable of enhancing gene expression in mouse embryonic stem cells (ESCs) [29]. Specifically, RLTR9B2, RLTR9D and RLTR9E subfamilies enrich for Esrrb, Kl4 and Sox2-binding motifs, in a specific organization and orientation. The approximation of the ancestral TE state (i.e. the RepBase sequence [30], which is based on a consensus-based approximation of the ancestral sequence) for these subfamilies also contains the same *cis*-regulatory grammar (arrangement and orientation of motifs), suggesting that the ancestral TEs spread these *cis*-regulatory binding modules (figure 1*c*) across the mouse genome, after the mouse–rat split less than 12–14 million years ago. Functional validation (using massively parallel reporter assays, MPRA [31]) of the modularity of these binding motifs reveals synergy (rather than merely additivity) to enhance gene expression in the ancestral TEs [13], as opposed to acquiring the regulatory potential via de novo mutations over evolutionary times [12]. Similarly, in human naive pluripotent cells, evolutionarily young, ape-specific LTR5HS and SVA elements are bound and regulated by pluripotency TFs OCT4 and KLF4 [32,33].

Early embryos express totipotency/pluripotency factors and have lower epigenomic restriction which provides a window of opportunity for transcriptional reactivation of TEs (figure 1*c*; [34–37]). Indeed, preimplantation embryos have widespread TE transcription, mediated by permissive chromatin and pluripotency TFs that bind to TEs. Once somatic gene expression programmes are switched on, epigenomic restriction increases and reduces TE activity. It is likely that ancestral TEs lack complete and optimized binding motifs for an entire repertoire of TFs that would be required for overcoming epigenomic restriction in a given somatic tissue, and instead in some instances gain these motifs via neutral substitution (figure 1*c*). In agreement with this notion, only a fraction of TEs within a specific class show active epigenomic states and TF-binding activity in somatic tissues, while most are silenced. Nonetheless, recent analyses of epigenomic states of TEs across 127 human cell types and tissues [10,38,39] revealed that TEs compose one-quarter of the regulatory epigenome (defined as active regulatory and transcribed chromHMM states, DNase hypersensitivity peaks and H3K27ac peaks) [40]. One emerging theme in these studies is that motif-rich LTRs of ERVs are particularly fruitful substrates for evolving new regulatory elements for the host, with many LTRs acquiring inducible or cell type-specific regulatory potential. Across the 127 human cell and tissue samples, almost half the TEs have a signature of putative active regulatory state in at least one of the analysed cell types. Interestingly, although TEs represent a significant fraction of regulatory and transcribed chromatin states across all epigenomes, in a given epigenome TEs are relatively depleted in active states, as most are silenced [40,41].

**Figure 2.**
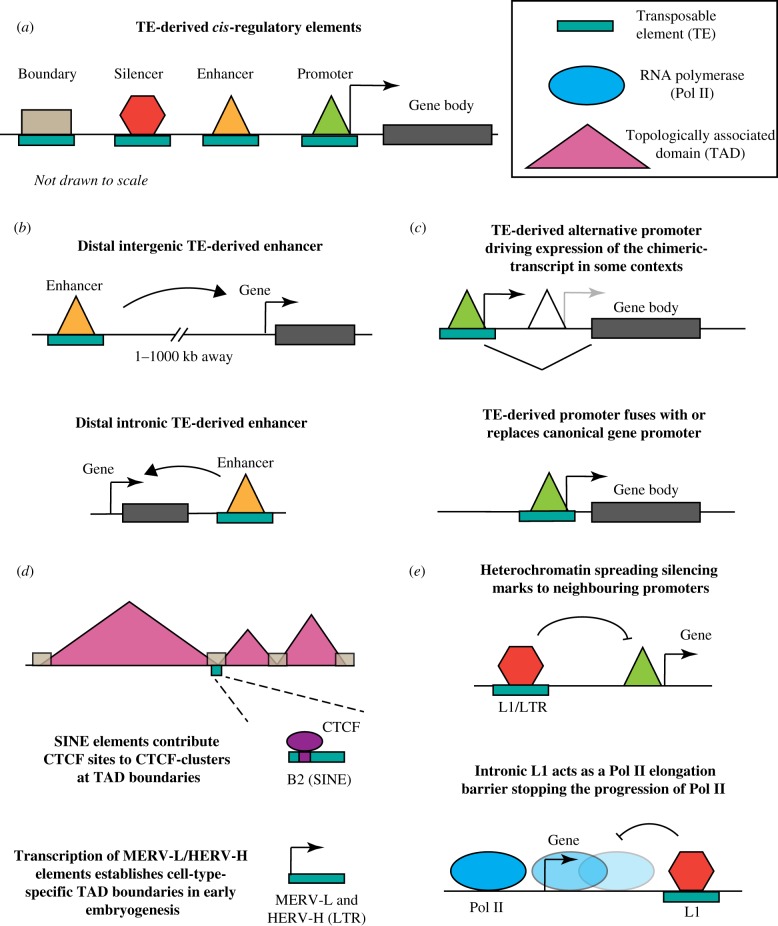
TE-derived *cis*-regulatory elements. (*a*) Here, we review TEs' role in gene expression via enhancers, promoters, boundary elements and silencers. (*b*) TE-derived enhancers act distally as either intergenic (upper panel) or intronic (lower panel). (*c*) TEs contribute promoters either as alternative promoters driving chimeric transcripts (upper panel) or as a replacement for the canonical promoter (lower panel). (*d*) TE-derived boundary elements contribute to topologically associated domains (TADs) by providing CTCF-binding sites (upper panel) and also maintain TADs by TE transcription (lower panel). (*e*) TEs can also act as a silencer by spreading heterochromatin (upper panel) or stalling Pol II elongation (lower panel).

*Cis*-regulatory elements such as enhancers and promoters (figure 2*a*) are densely populated clusters of TF-binding motifs which typically require occupancy of multiple TFs for their activation (reviewed in [42]). TEs that escape silencing and are bound by multiple TFs are much more likely to acquire features of the bona fide enhancer or promoter elements and in turn exert a functional impact on gene regulation. For example, the aforementioned mouse and human LTR elements bound by multiple pluripotency TFs are enriched for chromatin signatures associated with enhancers including H3K27ac and H3K4me1. More broadly, epigenomic surveys of different cell types and organisms found that a substantial portion of open chromatin regions are derived from TEs, including 63% of primate-specific regions [43]. Overall, TEs represent a potent source of context-specific regulators in the genome.

## 4. Transposable elements as a substrate for evolving new enhancers

Enhancers are canonically defined as non-coding DNA sequences that act to drive transcription independently of their relative distance, location or orientation to their cognate promoter (figure 2*b*). The ability of enhancers to act at a distance and in a modular and spatio-temporally restricted manner allows a gene to be regulated by multiple distal enhancers. This facilitates enormous combinatorial complexity of gene expression repertoires and allows the emergence of diverse phenotypic outcomes from the fixed set of genes [42,44–46]. As determinants of tissue-specific gene expression, enhancers can be considered as information integration hubs (reviewed in [47]) where genomic (i.e. sequence) information is read in the context of a specific cell type (by the lineage-specific TFs), signalling environment (by TF effectors of signalling pathways) and chromatin state (permissive or restrictive for TF access), and together is translated into precise spatio-temporal control of transcription. Over evolutionary times, enhancers are rapidly evolving regulatory elements compared with promoters and gene expression [48]. Indeed, developmental enhancers are fertile targets for evolutionary change, as they both are tissue-selective (allowing modulation of target gene transcription in a subset of tissues without affecting other pleiotropic gene functions) and commonly exist in groups of redundant or partially redundant elements (facilitating accumulation of genetic variation by buffering the risk of lethality). Thus, it comes as no surprise that TEs, which harbour TF-binding sites and can transpose to disseminate them across the genome, emerged as a major substrate for evolving tissue- and species-specific enhancers.

TE-derived enhancers are involved in context-specific gene regulation [47], affecting various biological cellular states, including pregnancy-related gene expression [21], early embryonic stages in humans [49] and innate immunity [20]. In the latter case, functional contribution of MER41 to STAT1-mediated interferon-γ-responsive enhancers and gene expression has been directly demonstrated [20]. Contributions of TEs to regulatory landscapes are particularly pronounced when species-specific enhancers are considered, including through waves of repeat expansions [12]. For example, the majority of ape-specific and human-specific liver enhancers overlap TEs (77% of ape-specific enhancers compared with 16% of evolutionarily conserved enhancers) [50]. Quantitative comparisons of enhancer landscapes between chimpanzee and human cranial neural crest cells (CNCCs) showed that species-specific changes in enhancer activity can be explained by the underlying regulatory sequence divergence and is linked to expression differences in genes involved in development of craniofacial structures [51]. Even when just orthologous human and chimpanzee sequences are considered (excluding species-specific TE insertions from the analysis), nearly half of the species-specific enhancers in CNCCs are derived from TEs and enriched for specific ERV and L1 families. Thus, TE sequences contribute towards enhancer evolution between closely related species and likely drive phenotypic differences too.

## 5. Transposable element origin of an hourglass developmental divergence?

Despite the key contribution of TEs to species-specific enhancers, in most mouse and human tissues TEs enriched for enhancer signatures are mostly ‘older’ than non-regulatory genomic TEs [40,41]. This suggests that mutations that occurred over evolutionary times allowed individual insertions within specific TE subclasses to acquire mutations facilitating gain of enhancer function (figure 1*a*). Indeed, uterine and liver enhancers have often evolved from ancient TE sequences [26,48]. Similarly, enhancer activity divergence between human and chimpanzee CNCCs commonly arises from just a few single-nucleotide mutations in older TE insertions [51]. Interestingly, this picture changes in preimplantation embryos and pluripotent cells, where evolutionarily young LTR elements, such as ape-specific LTR5HS in humans and murine-specific RLTR9B2, RLTR9D, RLTR9E and RLTR13D6 in the mouse, appear to have been broadly co-opted for enhancer function, with a large subset of individual elements within the given class showing the occupancy of pluripotency TFs (figure 1*c*) and chromatin signatures of active enhancers [29,52].

We speculate that such broad activity of evolutionarily young LTRs in naive pluripotency may not only result from permissive epigenetic state associated with global DNA hypomethylation, but may be a consequence of the fact that these ancient retroviruses must have been able to replicate in germ cells or preimplantation embryo cells in order to persist through vertical transmission (figure 1*c*). Thus, LTRs of retroviruses that successfully endogenized have likely been optimized for directing expression in early embryo/germ cells to begin with, and accumulating mutations over evolutionary times may decrease, rather than increase, their regulatory activity. One such example of a TE is HERV-H whose expression in human ESCs is not only abundant but also a pluripotency marker [53] and is mediated by pluripotency TFs [54,55]. Maladaptive insertions of such elements can be selected out of the population prior to implantation, whereas those that persist either are neutral or provide some adaptive benefits for the host. Interestingly, an hourglass model of developmental constraint postulates high regulatory divergence between closely related species in early and late development, and greater constraint at the phylotypic stage. It is tempting to speculate that the broad utilization of young (and often highly species-specific) TEs for regulation in the early embryo provides a major contribution to the species-specific differences in gene expression that have been observed during early embryogenesis [56–58].

Importantly, studies that perturb the function of these naive pluripotency-associated LTRs via CRISPR interference (CRISPRi) or LTR editing have found support for their contribution to long-range gene regulation [49,52]. Interestingly, however, effects of LTR5HS inactivation on gene expression in human cells are much more pervasive and stronger in magnitude that those seen for RLTR13D6 elements in mouse cells. Nearly 300 human genes, spanning a wide range of distances and distinct orientations with respect to the LTR, show significant dependency on the LTR5HS activity [49]. By contrast, only a minority RLTR13D5/RLTR13D6 elements seem to have a detectable effect on gene regulation in mouse ESCs [52]. Although a more robust CRISPRi approach used in the human study could potentially account for these disparate observations, it is also possible that genuine biological differences in inherent enhancer strength of the LTRs, distinct redundancies within regulatory landscapes or differences in selective pressures may account for diverse magnitude of LTR contributions to early embryonic gene regulation in apes and rodents. Regardless, these observations underscore the importance of functional assessment of regulatory contributions of specific TEs—or indeed, any candidate enhancer sequences—via perturbative experiments.

Over the recent years, since more systematic perturbative experiments of candidate non-coding sequences have become possible, we have learned that mammalian regulatory landscapes are extremely complex and often contain redundant enhancers and that the impact of individual putative enhancer deletion/silencing on expression of target genes is dependent on inherent enhancer strength, number and strength of other active enhancers acting on the same gene in a given cell type, and spatial constraints reflected in the frequency of contact with the target promoter(s) [59–63]. Furthermore, while relationships among enhancers within a regulatory landscape are often additive [64], examples of other relationships, including synergistic, competitive and hierarchical, have been described in the literature (reviewed in [42]). We should expect that TE-derived enhancers will participate in these complex relationships and thus their activity (or lack thereof) should be interpreted in a broader context of a given regulatory landscape.

## 6. Transposable element-derived promoters drive context-specific gene expression

Transcriptional promoters, like enhancers, consist of multiple TF-binding sites. While enhancers can act over very large genomic distances, promoters are immediately proximal to gene transcription start sites and contain *cis*-signals that allow efficient assembly of the Pol II preinitiation complex [45] (figure 2*c*). Although many promoters are bidirectional, most show a strong bias in the direction of transcription (of long stable transcripts), again in contrast with most enhancers, which give rise to bidirectional transcripts termed eRNAs (typically short and unstable) [45]. There is currently much debate in the field as to which functional features distinguish enhancers from promoters, and there appears to be a continuum, with a subset of elements able to function as both. In that context, it is interesting that LTR elements, which, by definition, are retroviral promoters, can also act as long-range enhancers for the host genes, independently of the transcriptional directionality or orientation of the LTR relative to the target promoter [49].

Nonetheless, LTRs in ERV sequences provide ready-made promoters in human and mouse [55,65–67]. TE-derived promoters [68] can either fuse with/replace a canonical gene promoter or serve as an alternative promoter either upstream or downstream from the canonical transcription start site (figure 2*c*). TE-derived alternative promoters can drive expression of chimeric transcripts, which may vary in levels when compared with those driven by the canonical promoter (figure 2*c*; reviewed in [69]). In some cases, such transcripts can also differ in their coding potential, giving rise to either chimeric or truncated proteins, and thus facilitating evolutionary innovation at the protein level [69]. Around 18% of human transcription start sites are defined by CAGE-seq overlap TEs [70], and tend to be in gene-dense regions often functioning as alternative promoters [71]. The first well-characterized example of TE-derived promoters was found in viable yellow agouti (A^vy^) mice, where the A^vy^ allele contains an IAP (intracisternal A particle; LTR) element in the 5′ end of the *agouti* (A) allele [72]. Variable DNA methylation levels across individuals at the A^vy^ promoter result in a variable coat colour in each mouse, in addition to other traits [73], and can also be influenced by maternal diet [74,75]. In the totipotent two-cell stage (2C) of mouse embryos and in 2C-like cells which spontaneously arise in mouse ESC cultures, MERVL elements are bound by TF Dux and serve as alternative promoters, giving rise to chimeric transcripts of the so-called 2C genes [76,77]. The human genome also encodes context-specific TE-derived promoters. For example, more than a thousand human genes have promoters derived from TEs [78,79]. LTRs among these TE-derived promoters enrich for cell-specific gene regulation. Epigenetic modification by deleting DNA methyltransferases in human neuronal progenitor cells results in specific activation of young, hominoid-specific LINE-1 (L1) elements [80], which can then serve as alternative promoters for neuronal protein-coding genes.

LINE-L1 (i.e. Long INterspersed Elements; Class I autonomous TEs) elements are also capable of bidirectional transcription: L1s have two promoters in their 5′ UTR—one sense and one antisense [81]. L1 antisense promoters produce many chimeric transcripts from adjacent genes [82]. Disease conditions that alter epigenetic landscape can promote aberrant activation of promoters contained within TEs. Recent work analysing transcripts in thousands of cancer samples (from The Cancer Genome Atlas—TCGA—Program) demonstrates are epigenetic reactivation of cryptic promoters in TEs that can drive gene expression in cancer, often referred to as onco-exaptation ([27,83]; examples are listed in [69]). TE-derived onco-exaptation events are not only responsible for a significant fraction of the target oncogene's expression in the examined samples but were also associated with poor patient survival. Taken together, TE-derived gene promoters can impact both developmental and diseased transcription states.

## 7. Role of transposable elements in three-dimensional genome architecture

In addition to sequences that promote or enhance gene expression, TEs also contribute to the maintenance of genome architecture. Three-dimensional chromatin organization not only determines the packaging of DNA in the cell, but also influences gene regulation by demarcating regulatory neighbourhoods (figure 2*d*). Measurements of chromosome contacts (from a genome-wide chromosome conformation capture assay—Hi-C, as reviewed in [84]) identified self-associating domains known as topologically associating domains (TADs; [85,86]). TAD boundaries are enriched for binding of CTCF, a zinc finger protein that mediates the formation of structural chromatin loops by restraining cohesin-mediated loop extrusion [86–88]. SINE elements are enriched for CTCF binding [9,11], and are overrepresented at TAD boundaries [85]. However, TAD boundaries are typically deeply conserved in evolution [89], suggesting that species-specific rewiring of TADs by TEs is not common. Instead, it has been suggested that rodent SINEs can contribute to the maintenance of clustered CTCF sites at TAD boundaries, which in turn promotes the maintenance of genome organization [90]. Clusters of CTCF sites are over-represented at TAD boundaries and likely provide buffering against turnover of individual sites to stabilize TADs and chromatin loops. Along with maintaining chromatin contact loops through TE-derived CTCF sites, TEs can also contribute to the establishment of species-specific chromatin loops by depositing novel anchor CTCF motifs [91].

Although most TAD-boundary elements are cell-type invariant, evolutionarily conserved and enriched for CTCF motifs, a small subset of TAD boundaries appears to escape this generality [92]*.* Indeed, a recent report highlights pluripotent stem-cell-specific TAD boundaries that are detected in human ESCs, but weakened during differentiation to cardiomyocytes [93]. These cell-type-specific boundaries are devoid of clusters of CTCF sites, and instead enriched in highly transcribed HERV-H elements, a primate-specific class of LTR TEs with previously described roles in pluripotency [53,54]. The establishment of TAD boundaries is dependent on the transcription of HERV-H (figure 2*d*), as directed silencing of the HERV-H can eliminate the insulation and impact the gene regulatory landscape. Similarly, in mouse 2C cells, MERVL elements are not only the main source of promoters for directing expression of the early 2C genes, but also facilitate the formation of chromatin domain boundaries in a manner that coincides with their transcriptional upregulation [94]. Intriguingly, these new observations again highlight a particularly prevalent role for TEs in genome regulation and organization during early development.

## 8. Collateral effects of transposable element silencing on gene regulation

Although TEs provide a rich substrate for evolving diverse *cis*-regulatory elements for the host, most genomic TEs are epigenetically silenced to prevent mutagenic effects of their transposition. These silencing events often occur within transcriptional gene units, as a large subset of TEs (estimated at approx. 60% in the human and mouse genomes) is located in introns [95]. For genes that are active, this can lead to conflicts between polymerase passage through the gene and heterochromatin formation at intronic or promoter-proximal TEs, and in turn can affect host gene expression (figure 2*e*). One feature of heterochromatin is its ability to spread beyond the initial recruitment site via mechanisms that often involve coupling of the epigenetic writers and readers (reviewed in [96]). For example, in tetrapod vertebrates, KRAB zinc finger proteins mediate the repression of specific TE subclasses via DNA-sequence recognition and recruitment of a repressive complex including H3K9me3 methyltransferase SETDB1 and the reader of H3K9me3, HP1 [97,98]. KRAB-domain-mediated silencing typically spreads to several, and in some cases even tens of kilobases away from the initial recruitment site, which can lead to the repression at neighbouring gene promoters [99,100]. Similarly, DNA methylation can spread from TEs to nearby promoters, although some studies suggest that such events are relatively rare and actively counteracted by the host [100,101].

Interestingly, TE silencing events can impact gene expression even over large genomic distances from promoters. For example, evolutionarily younger, full-length LINE-1 (L1) elements located within introns of active genes are preferentially targeted for repression and H3K9me3-deposition by the human silencing hub (HUSH) complex [102,103]. From the genome defence perspective, such elements—encompassing transposition-competent L1PA1 (L1Hs)—need to be suppressed as they pose the highest threat to genomic integrity, especially when embedded within a transcriptionally permissive environment. However, these intronic silencing events create heterochromatic islands of at least 7 kb length (the length of a full-length L1) that elongating Pol II must pass through, and genic H3K9me3 as well as L1 insertions have both been shown to decrease Pol II elongation rates [104,105]. Consequently, transcript levels of active genes containing evolutionarily young full-length L1s are downregulated, often subtly, in an L1 sequence-dependent and HUSH complex-dependent manner [102]. These L1s can be thought of as genetically determined but epigenetically regulated silencer elements that can quantitatively modulate expression levels of hundreds of human genes at large distances from gene promoters. Thus, the host genome needs to balance the need for young intragenic TE suppression with the collateral effects that such silencing can have on gene expression.

## 9. Concluding remarks

Evolutionary differences across species are thought to be largely driven by changes in gene expression, mediated by divergence in *cis*-regulatory elements [106,107]. Recent progress in the field revealed that a substantial portion of mammalian *cis*-regulatory sequences is derived from TEs. These TE-derived *cis*-regulatory elements are often cell type- and species/clade-specific and can contribute to gene expression regulation through many diverse mechanisms, which we have reviewed here.

The prevalence of TE utilization for regulatory functions may differ between cell types and developmental stages. Indeed, TEs seem to play an outsized role during mammalian pre- and peri-implantation development, where whole subclasses of TEs (such as MERVL in mice, or HERV-K and HERV-H in humans) function in host gene regulation as alternative promoters, enhancers or boundary elements. This widespread utilization of TEs during early development is likely facilitated both by the global epigenomic de-repression during this time of embryogenesis and by the fact that to successfully propagate through vertical transmission, ancestral TEs must have been able to direct transcription of their own sequences in toti/pluripotent cells or germ cells. Consequently, many evolutionarily young TEs, which are typically species- or clade-specific, can bind toti- and pluripotency-associated TFs. We speculate that, at least in mammals, transcriptional divergence during early development, as postulated by the hourglass model, is largely mediated by the massive co-option of TEs for host gene regulation.

By contrast, in somatic tissues, epigenomic control is restored and TEs are largely repressed via a multitude of chromatin-silencing mechanisms. However, different fractions of TEs, often representing older insertions, escape the epigenomic control by accumulating sequence changes that make them compatible with cell-type- and context-specific functions. Since we have only begun to systematically assess the function of TEs in gene control, it is likely that we are still vastly underestimating their impact as well as the diversity of mechanisms by which TEs can influence transcription, post-transcriptional gene regulation, genome organization and evolutionary divergence. Nevertheless, our understanding of TEs has come a long way from the notion of ‘junk’ DNA. What persists is Barbara McClintock's early vision of TEs as ‘controlling elements' [108].

## Data accessibility

This article has no additional data.
